# Hemorrhagic retinal arterial macroaneurysm combined with branch retinal artery occlusion treated with intravitreal conbercept injection: A case report

**DOI:** 10.1097/MD.0000000000035434

**Published:** 2023-10-27

**Authors:** Pinxue Xie, Wen Feng, Ran Yan, Siquan Zhu, Xinxiao Gao

**Affiliations:** a Beijing Institute of Heart, Lung and Blood Vessel Diseases, Beijing, China; b Department of Ophthalmology, Beijing Anzhen Hospital, Capital Medical University, Beijing, China.

**Keywords:** branch retinal artery occlusion, conbercept, retinal arterial macroaneurysm

## Abstract

**Rationale:**

Branch retinal artery occlusion (BRAO) is a rare complication of retinal arterial macroaneurysm (RAM), a low-incidence ocular disease.

**Patient concerns:**

A 75-year-old woman presented with a chief complaint of blurred vision.

**Diagnoses:**

The patient for 4 days received a diagnosis of RAM combined with BRAO.

**Interventions:**

The patient was treated with two successive intravitreal conbercept injections.

**Outcomes:**

The patient’s best-corrected visual acuity improved, and the RAM diminished.

**Lessons:**

Administration of conbercept injection might be an effective treatment for complex RAM with BRAO.

## 1. Introduction

With a prevalence of 1/9000 in China and 1/4500 worldwide, retinal arterial macroaneurysm (RAM) frequently affects women aged 60 to 80 years.^[1]^ RAM commonly occurs in 1 eye, with a 10% prevalence in both eyes.^[2]^ Most RAMs are located within tertiary branches of the retinal artery, particularly in the superior temporal branches.^[3]^ Most RAM patients are asymptomatic and do not require special treatment. However, some patients may develop retinal exudates, macular edema, and other complications causing severe visual loss. Branch retinal artery occlusion (BRAO) is a rare complication of RAM.^[4,5]^ Successful treatment of such cases has not been reported yet.

Conbercept (Chengdu Kanghong Biotech Co., Ltd., Sichuan, China) is a novel recombinant fusion protein with a high affinity for all vascular endothelial growth factor (VEGF) isoforms.^[6,7]^ Conbercept, a potent VEGF inhibitor, has been approved to protect against various retinal vasculopathies, including wet age-related macular degeneration, diabetic macular edema, myopic choroidal neovascularization, retinal vein occlusion, etc.^[8,9]^ This study reports a case of RAM combined with BRAO that was treated successfully with intravitreal conbercept injection.

## 2. Case presentation

**Figure 1.**
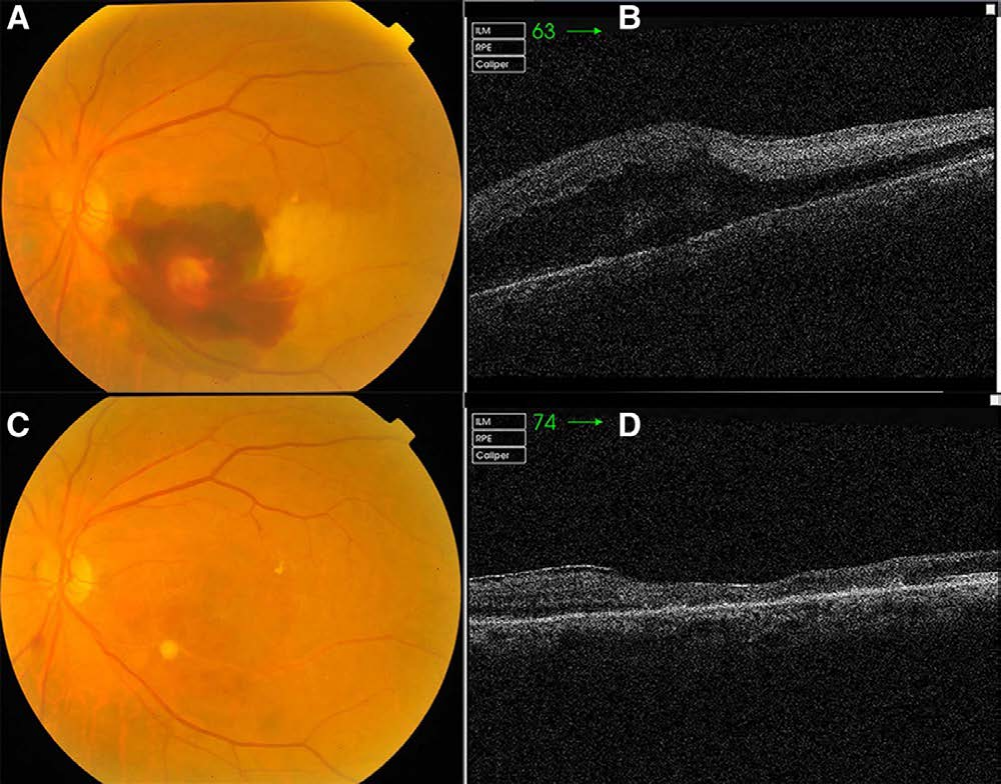
Patient preoperative and postoperative findings. (A) Before intravitreal injection, a large retinal arterial macroaneurysm in the inferior-temporal branch artery around 2 papillary diameters away from the optic disk, with peripheral subretinal and preretinal hemorrhage involving the macular center. (B) Spectral-domain optical coherence tomography (SD-OCT) revealed intraretinal and subretinal fluid in the macula of the left eye. (C) After 2 intravitreal conbercept injections, the macroaneurysm diminished with approximately complete absorption of the subretinal and preretinal hemorrhage. (D) SD-OCT indicated that the subretinal hemorrhage was absorbed and the distal retinal edema was relieved, with partial retinal atrophy and epiretinal membrane formation.

**Figure 2.**
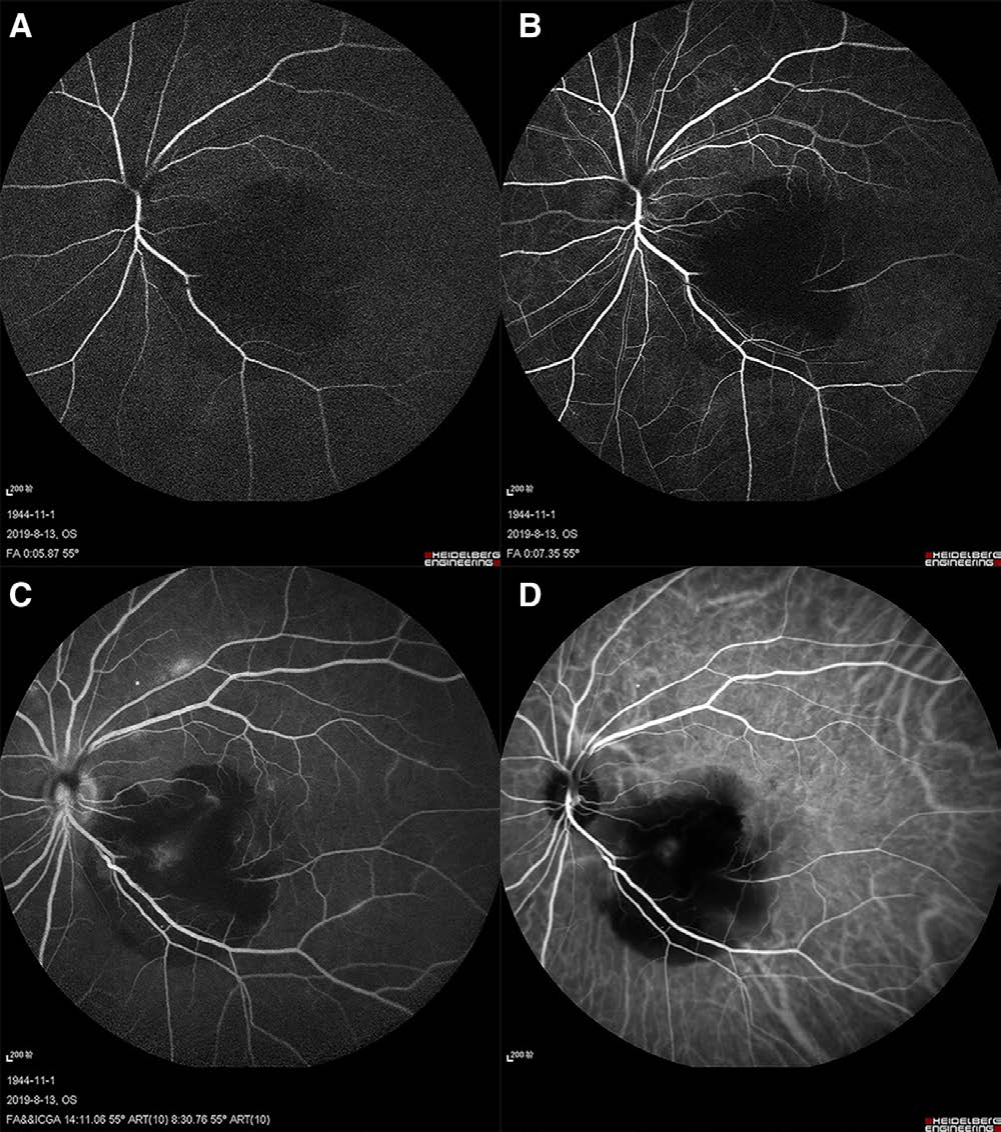
Fluorescence angiography shows (A) and (B). the inferior-temporal branch artery distal to the retinal arterial macroaneurysm (RAM) was filling sluggishly, and the filling fronts were visible in the early stage. (C) Fluorescein leaked around the aneurysm in the late stage in the left eye. (D) The morphology of the RAM was more clearly visible on indocyanine green angiography (ICGA) than that on fundus fluorescein angiography. ICGA showed a well-defined focal area of hypercyanescence, which increased in intensity over time.

This is the case of a 75-year-old woman who presented to our outpatient clinic with a complaint of blurred vision in her left eye for 4 days. The patient had a history of hypertension, diabetes mellitus, and osteoarthrosis for over 10 years. The patient had been diagnosed with latent coronary heart disease a year earlier and was being treated with aspirin for this chronic cardiopathy. Her best-corrected visual acuity (BCVA) was 20/30 in the right eye and counting fingers in the left eye. The intraocular pressure was 16 mmHg for both eyes. The anterior segment examination findings were unremarkable; however, mild lens clouding was noted in both eyes. Fundoscopic examination revealed a large RAM in the inferior-temporal branch artery, located approximately 2 papillary diameters away from the optic disc, with peripheral subretinal and preretinal hemorrhages involving the macular center. Thinning was observed in the retinal artery distal to the aneurysm, with surrounding retinal grayish edema (Fig. 1A). Spectral-domain optical coherence tomography revealed intraretinal and subretinal fluid in the macula of the left eye (Fig. 1B). In the early stage, the inferior-temporal branch artery distal to the RAM was filling sluggishly, and the filling fronts were visible (Figs. 2A and 2B); however, in the late stage, fluorescein leaked near the aneurysm in the left eye, as observed through fluorescence angiography (Fig. 2C). RAM morphology was visible with a well-defined focal area of hypercyanescence, which increased in intensity over time on indocyanine green angiography (Fig. 2D). Based on the aforementioned observations, the following diagnoses were made: RAM in the left eye, inferior-temporal BRAO, and bilateral age-related cataracts.

After obtaining informed consent from the patient and her legal guardian, the left eye was treated with an intravitreal conbercept injection (0.5 mg) on August 15, 2019. Two weeks after the first injection, subretinal hemorrhage was partially absorbed, and the distal retinal edema was alleviated. On September 26, after communicating with the patient, the second intravitreal conbercept injection was administered. At a 3-month follow-up after the second injection, the RAM had diminished, and the subretinal and preretinal hemorrhages completely disappeared (Fig. 1C). Furthermore, the left eye had an improved BCVA of 20/200. Spectral-domain optical coherence tomography showed partial retinal atrophy and epiretinal membrane formation (Fig. 1D).

## 3. Discussion and conclusions

Cases of RAM combined with BRAO are rarely reported, and according to Robert et al, the natural prevalence of such cases is only 8%.^[4,5]^ This study reports a case of an elderly female patient with a RAM complicated by BRAO presenting with a subretinal hemorrhage involving the macular center. The occurrence of RAM is associated with atherosclerosis, hypertension, and dyslipidemia.^[2]^ BRAO and RAM might have similar pathogenic factors of BRAO and RAM, including damaged local vascular endothelium and local vessel wall weakness; may cause vessel wall dilatation and plaque formation.^[10,11]^ In this case, existing hypertension and coronary heart disease, vessel wall degeneration, loss of vascular regulation, and arterial dilatation with local platelet aggregation in the vessel might have led to RAM complicated by BRAO.^[10]^

Unruptured RAM is self-limiting; however, complications related to vision loss warrant medical treatment.^[12]^ In this case, the patient had ruptured RAM involving the macular fovea. For ruptured RAM, the primary treatment modalities include laser photocoagulation, intravitreal injection of anti-vascular endothelial growth factor (anti-VEGF), anti-VEGF injection combined with laser photocoagulation, Nd:YAG laser treatment, and pars plana vitrectomy.^[2,13]^ Direct photocoagulation may aid the closure of large aneurysms, while retinal laser treatment might lead to complications including choroidal neovascularization, laser scarring, and retinal artery occlusion.^[2,14,15]^ After laser photocoagulation of RAM, BRAO prevalence is as high as 16%.^[4,5]^ Consequently, in this case, retinal photocoagulation was not used. Intravitreal injection of anti-VEGFs, including ranibizumab, bevacizumab, and aflibercept, is a novel treatment method and has excellent therapeutic effects on RAM with macular edema.^[2–4]^ Compared with aflibercept, conbercept has an additional 4th binding domain of VEGF-R2, which may further extend its clearance time.^[6,7]^ Lin et al reported a case of RAM, wherein the macular hemorrhage, edema resolution, and BCVA improved after a single conbercept injection.^[16]^ Anti-VEGF injections reduce nitric oxide production by vascular endothelial cells and cause vasoconstriction, thereby decreasing macular edema. In this case, the patient exhibited almost complete absorption of subretinal hemorrhage after 2 intravitreal conbercept injections, presumably because the anti-VEGF therapy improved the equilibrium between coagulation and fibrinolysis and then accelerated the absorption of the subretinal hemorrhage.^[2]^ However, the patient had coronary heart disease and was being treated with aspirin, which may have caused the patient RAM rupture hemorrhage. The patient continued to have aspirin during eye treatment for hemorrhage, which may have promoted complete absorption of blood from the retina.^[17]^ Upon searching the PubMed database, this study is the first to report a case of RAM combined with BRAO and its successful treatment with the anti-VEGF injection. The final visual recovery was not achieved, although the RAM subsided, the central retinal thickness decreased, and the anatomical structure remained intact. One of the primary causes of poor visual recovery may be iron released from the subretinal hemorrhage that exhibits a toxic effect and causes oxidative stress on the photoreceptor cells and RPE. The presence of BRAO in patients with retinal ischemia may also be one of the causes of retinal structural damage and reduced macular thickness.^[18]^ Moreover, the hemorrhage barrier effect may have affected the inner retina nutrient and metabolic processes.^[5]^ Furthermore, the patient had developed epiretinal membranes with retinal tissue atrophy, which have contributed to poor visual acuity. Drugs targeting the Ang/Tie and VEGF signaling pathways to treat hemorrhagic retinal diseases, including faricimab, are under evaluation in clinical trials. These novel drugs may stabilize RAM structure and reduce its permeability; thus, providing a promising treatment method for these patients.^[19,20]^ In addition, privious studies have proved the efficacy of the intraocular administration of tissue plasminogen activator with anti-VEGF drugs for submacular hemorrhage, which can significantly reduce foveal thickness and improve BCVA.^[21,22]^ This technique might be attempted and evaluated in cases like ours in the future. In conclusion, BRAO is a rare complication of RAM that can be effectively treated with the intravitreal conbercept injection.

## Author contributions

**Conceptualization:** Wen Feng, Xinxiao Gao.

**Data curation:** Ran Yan, Siquan Zhu.

**Formal analysis:** Pinxue Xie.

**Project administration:** Siquan Zhu, Pinxue Xie, Ran Yan.

**Supervision:** Xinxiao Gao.

**Validation:** Xinxiao Gao.

**Writing – original draft:** Pinxue Xie.

**Writing – review & editing:** Xinxiao Gao.

## References

[1] Xu L Wang Y Jonas JB. Frequency of retinal macroaneurysms in adult Chinese: the Beijing Eye Study. Br J Ophthalmol. 2007;91:840–1. 17510482 10.1136/bjo.2006.107342 PMC1955572

[2] Chatziralli I Maniatea A Koubouni K. Intravitreal ranibizumab for retinal arterial macroaneurysm: long-term results of a prospective study. Eur J Ophthalmol. 2017;27:215–9. 27646333 10.5301/ejo.5000863

[3] Mansour AM Foster RE Gallego-Pinazo R. Intravitreal anti-vascular endothelial growth factor injections for exudative retinal arterial macroaneurysms. Retina. 2019;39:1133–41. 29505440 10.1097/IAE.0000000000002131

[4] Panton RW Goldberg MF Farber MD. Retinal arterial macroaneurysms: risk factors and natural history. Br J Ophthalmol. 1990;74:595–600. 2285682 10.1136/bjo.74.10.595 PMC1042226

[5] Lavin MJ Marsh RJ Peart S. Retinal arterial macroaneurysms: a retrospective study of 40 patients. Br J Ophthalmol. 1987;71:817–25. 3689733 10.1136/bjo.71.11.817 PMC1041318

[6] Liu K Song Y Xu G. Conbercept for treatment of neovascular age-related macular degeneration: results of the randomized phase 3 PHOENIX study. Am J Ophthalmol. 2019;197:156–67. 30148987 10.1016/j.ajo.2018.08.026

[7] Ferro Desideri L Traverso CE Nicolò M. An update on conbercept to treat wet age-related macular degeneration. Drugs Today (Barc). 2020;56:311–20. 32406878 10.1358/dot.2020.56.5.3137164

[8] Zhang J Liang Y Xie J. Conbercept for patients with age-related macular degeneration: a systematic review. BMC Ophthalmol. 2018;18:142. 29902977 10.1186/s12886-018-0807-1 PMC6003117

[9] Liu H Ma Y Xu HC. Updates on the management of ocular vasculopathies with VEGF inhibitor conbercept. Curr Eye Res. 2020;45:1467–76. 32631094 10.1080/02713683.2020.1781193

[10] Lewis RA Norton EW Gass JD. Acquired arterial macroaneurysms of the retina. Br J Ophthalmol. 1976;60:21–30. 1268157 10.1136/bjo.60.1.21 PMC1017462

[11] Abu-El-Asrar AM. Retinal arterial macroaneurysm at the site of a retinal artery embolus. Eye (Lond). 2001;15(Pt 5):655–7. 11702980 10.1038/eye.2001.203

[12] Speilburg AM Klemencic SA. Ruptured retinal arterial macroaneurysm: diagnosis and management. J Optom. 2014;7:131–7. 25000868 10.1016/j.optom.2013.08.002 PMC4087178

[13] Chen YY Lin LY Chang PY. Laser and anti-vascular endothelial growth factor agent treatments for retinal arterial macroaneurysm. Asia Pac J Ophthalmol (Phila). 2017;6:444–9. 28828763 10.22608/APO.201766

[14] Kitagawa Y Kawamorita A Shimada H. Treatment of macular hemorrhage in retinal arterial microaneurysm: anatomic site-oriented therapy. Jpn J Ophthalmol. 2019;63:186–96. 30783941 10.1007/s10384-019-00653-y

[15] Russell SR Folk JC. Branch retinal artery occlusion after dye yellow photocoagulation of an arterial macroaneurysm. Am J Ophthalmol. 1987;104:186–7. 3618718 10.1016/0002-9394(87)90015-8

[16] Lin Z Hu Q Wu Y. Intravitreal ranibizumab or conbercept for retinal arterial macroaneurysm: a case series. BMC Ophthalmol. 2019;19:18. 30646868 10.1186/s12886-019-1035-z PMC6334469

[17] Yang JF Kishore K. Recurrent vitreous hemorrhage from an optic nerve retinal arterial macroaneurysm. Case Rep Ophthalmol. 2017;8:503–9. 29282401 10.1159/000481704 PMC5731140

[18] Leung CK Tham CC Mohammed S. In vivo measurements of macular and nerve fibre layer thickness in retinal arterial occlusion. Eye (Lond). 2007;21:1464–8. 16751755 10.1038/sj.eye.6702457

[19] Ferro Desideri L Traverso CE Nicolò M. The emerging role of the Angiopoietin-Tie pathway as therapeutic target for treating retinal diseases. Expert Opin Ther Targets. 2022;26:145–54. 35098845 10.1080/14728222.2022.2036121

[20] Nicolò M Ferro Desideri L Vagge A. Faricimab: an investigational agent targeting the Tie-2/angiopoietin pathway and VEGF-A for the treatment of retinal diseases. Expert Opin Investig Drugs. 2021;30:193–200. 10.1080/13543784.2021.1879791 33471572

[21] Stanescu-Segall D Balta F Jackson TL. Submacular hemorrhage in neovascular age-related macular degeneration: a synthesis of the literature. Surv Ophthalmol. 2016;61:18–32. 26212151 10.1016/j.survophthal.2015.04.004

[22] He X Cao W Wang Z. Efficacy evaluation of tissue plasminogen activator with anti-vascular endothelial growth factor drugs for submacular hemorrhage treatment: a meta-analysis. J Clin Med. 2023;12:1035. 36769682 10.3390/jcm12031035 PMC9918283

